# Txnip regulates the Oct4-mediated pluripotency circuitry via metabolic changes upon differentiation

**DOI:** 10.1007/s00018-024-05161-y

**Published:** 2024-03-15

**Authors:** Sojung Kwak, Cho Lok Song, Yee Sook Cho, Inpyo Choi, Jae-Eun Byun, Haiyoung Jung, Jungwoon Lee

**Affiliations:** 1 Developmental Biology Laboratory, Environmental Disease Research Center, Korea Research Institute of Bioscience and Biotechnology, Daejeon, 34141 Republic of Korea; 2 Stem Cell Research Laboratory, Immunotherapy Research Center, Korea Research Institute of Bioscience and Biotechnology, Daejeon, 34141 Republic of Korea; 3 Department of Bioscience, KRIBB School, University of Science and Technology, Daejeon, 34141 Republic of Korea; 4 Immunotherapy Research Center, Korea Research Institute of Bioscience and Biotechnology, Daejeon, 34141 Republic of Korea; 5 Aging Convergence Research Center, Korea Research Institute of Bioscience and Biotechnology, Daejeon, 34141 Republic of Korea; 6 Department of Biochemistry, School of Life Sciences, Chungbuk National University, Cheongju, 28644 Republic of Korea

**Keywords:** Thioredoxin interacting protein (Txnip), Pluripotent stem cells (PSCs), Reprogramming, Pluripotency, Differentiation, Glycolysis, Histone acetylation, Oct4

## Abstract

Thioredoxin interacting protein (Txnip) is a stress-responsive factor regulating Trx1 for redox balance and involved in diverse cellular processes including proliferation, differentiation, apoptosis, inflammation, and metabolism. However, the biological role of Txnip function in stem cell pluripotency has yet to be investigated. Here, we reveal the novel functions of mouse Txnip in cellular reprogramming and differentiation onset by involving in glucose-mediated histone acetylation and the regulation of Oct4, which is a fundamental component of the molecular circuitry underlying pluripotency. During reprogramming or PSC differentiation process, cellular metabolic and chromatin remodeling occur in order to change its cellular fate. *Txnip* knockout promotes induced pluripotency but hinders initial differentiation by activating pluripotency factors and promoting glycolysis. This alteration affects the intracellular levels of acetyl-coA, a final product of enhanced glycolysis, resulting in sustained histone acetylation on active PSC gene regions. Moreover, Txnip directly interacts with Oct4, thereby repressing its activity and consequently deregulating Oct4 target gene transcriptions. Our work suggests that control of Txnip expression is crucial for cell fate transitions by modulating the entry and exit of pluripotency.

**Supplementary Information:**

The online version contains supplementary material available at 10.1007/s00018-024-05161-y.

## Introduction

Pluripotent stem cells (PSCs), such as embryonic stem cells (ESCs) and induced pluripotent stem cells (iPSCs), have a unique metabolic process and reduction–oxidation (redox) state to maintain their pluripotency [1, 2]. PSCs rely on glycolysis metabolism, which produces reduced reactive oxygen species (ROS) [2]. Thus, low ROS levels are essential for the PSC maintenance, whereas oxidative stress due to increased ROS production leads to a shift in PSC fate toward differentiation [3]. Indeed, a metabolic switch from glycolysis to oxidative phosphorylation (Oxphos) occurs upon PSC differentiation. Conversely, during somatic cell reprogramming, a metabolic shift from OxPhos to glycolysis affects the efficient iPSC generation [3]. Glycolysis enhanced by reprogramming into iPSCs produces upregulated levels of acetate and acetyl-CoA, which are known substrates for acetylation. Conversely, early differentiation of PSCs results in a rapid loss of glycolysis, which decreases acetyl-CoA and acetate levels, causing a reduction in histone acetylation and consequently leading to pluripotency exit [4]. Therefore, tight control of a balanced metabolic and redox state is essential to maintain the pluripotent state of stem cells. Interestingly, the core pluripotency factors, Oct4, Sox2, and Nanog, are involved in the regulation of glucose metabolism of PSCs [5]. In particular, Oct4, a fundamental pluripotency factor, is also a critical regulator of glycolysis by directly activating the expression of glycolytic enzymes, especially Hk2 and Pkm2 [6]. Such a distinct molecular link between pluripotency regulators and metabolic features needs to be further investigated in controlling PSC fate determination.

Thioredoxin interacting protein (Txnip) is a pro-oxidant protein which has an α-arrestin domain that interacts with Thioredoxin (Trx), thereby reducing Trx activity. As a repressor of Trx, Txnip is known to increase ROS production and induce oxidative stress, thereby controlling cellular redox signaling [7, 8]. In addition, Txnip controls glucose uptake by regulating glucose transporter 1 (Glut1) expression and localization [9] and integrates energy metabolism and growth regulation by modulating PTEN and Akt activation in oxidative tissues [10]. As we have previously reported, Txnip deficiency in type 1 diabetic mice efficiently reduces blood glucose levels and ultimately increases mice survival, suggesting that Txnip may be a potential therapeutic target for the development of diseases such as diabetes [11]. Moreover, Txnip knock-out (KO) mouse embryonic fibroblasts (MEFs) are known to induce higher glucose uptake than wild-type (WT) MEFs [11], but it is still unknown whether Txnip affects the reprogramming of MEFs into iPSCs and/or the conversion of PSC fate. Therefore, it is necessary to investigate whether Txnip regulates a transcriptional regulatory network in pluripotency and differentiation by a fine-tuning of cellular stress.

In this study, we found that mouse Txnip deficiency induces cellular reprogramming through earlier activation of core pluripotency factors. However, enriched stemness genes in *Txnip* KO iPSCs retards the exit from pluripotency and the progress of differentiation. To scrutinize this phenomenon, we performed transcriptome analysis, which revealed that Txnip regulates multiple biological processes related to organ morphogenesis, development, angiogenesis, and metabolic processes. Txnip loss not only induces the expression of pluripotency genes but also increases glycolytic enzymes, by sustaining a high level of glycolytic flux resulting in a higher level of acetyl-coA, an ingredient for histone acetylation. Furthermore, biochemical analysis demonstrates that Txnip interacts with Oct4 as a transcriptional corepressor to regulate Oct4-dependent gene expression, revealing a role for the Txnip-Oct4 complex in defining the onset of differentiation in PSCs.

## Materials and methods

### iPSC reprogramming

WT and *Txnip* KO MEFs were derived from E13.5 mouse embryos and cultured in MEF medium (high glucose DMEM, 20% FBS, and 100 U penicillin and streptomycin). MEFs were seeded and delivered with competent sendai virus (SeV) that carry Yamanaka factors using CytoTune-iPS Sendai Reprogramming (Theromo) to reprogram MEFs into iPSCs, as previously reported [12, 13]. iPSC colonies were picked and expanded for further iPSC characterization. PSC medium was composed of Dulbecco’s modified Eagle’s medium (DMEM) (Gibco) supplemented with Fetal bovine serum (FBS) (15%, Gibco), Glutamax™ supplement (1%, Gibco), MEM Non-Essential Amino Acids (1%, Gibco), penicillin/streptomycin (1%, Gibco) and ESGRO recombinant mouse LIF (1000 U/ml, Millipore).

### Cell culture

Mouse PSCs were mainly cultured in high glucose DMEM (Gibco, 11965092) supplemented with 15% FBS (Gibco), Glutamax™ supplement (1%, Gibco), β-mercaptoethanol (55 μM, Gibco), MEM non-essential amino acid (1%, Gibco), penicillin and streptomycin (1%, Gibco), and ESGRO recombinant mouse LIF (1000 U/ml, Millipore), as previously reported [14]. For low glucose conditions, high glucose DMEM (Gibco, 11965092) was mixed with no glucose DMEM (Gibco, 11966025) in 1:4 ratio, generating final glucose concentration of 5 mM. Spontaneous differentiation of PSCs was induced by LIF withdrawal from PSC medium and addition of retinoic acid (RA, final concentration 0.5 µM) in monolayer cultures. For embryonic body (EB) formation, PSCs were cultured in low-attachment dishes that contained PSC medium without LIF. Briefly, PSCs were trypsinized and cultivated as EBs by hanging drop method (500 cells/10 µl drop) in the absence of LIF for 2 days followed by 4-day suspension culture.

### AP staining

AP staining was done as described [14, 15] using an AP detection kit (Sigma-Aldrich). Briefly, reprogrammed cells were fixed and stained with AP staining solution in the dark. PSCs were plated at a density of 1000 cells/well in a 6-well plate and incubated for 5 to 6 days. AP-positive colonies were counted and radius of each colony was measured.

### Lentiviral shRNA-mediated knock-down

pLKO.1 lentiviral vectors for shRNAs (TRCN0000347096 and TRCN0000182360) (Sigma-Aldrich) were purchased for knock-down experiments. 293FT cells plated on 6 well plates were used for lentivirus production. Each well was co-transfected with 0.5 μg each of pMD2.G, pMDLg/pRRE, pRSV-rev, and pLKO.1-shRNA using Lipofectamine 2000 (Invitrogen). 48 h after transfection, the virus-containing medium was collected and filtered through 0.45 μm filters. Polybrene (8 μg/ml) was added just before target cell infection. The viral infection was performed for 6 h. Media were changed with fresh PSC media afterwards. 48 h post infection, puromycin selection (2 μg/ml) was performed for a minimum of 4 days. Lentiviral shRNA-mediated Txnip knock-down was validated by qRT-PCR and western blot with Txnip specific antibody and primer.

### Generation of stable PSC lines

Full-length mouse *Txnip* cDNAs were obtained from J1 ESC cDNA by PCR and further cloned into the pCAG-Flag vector. Stably expressed Flag-tagged Txnip iPSCs were generated by introducing the pCAG-Flag-Txnip into WT iPSCs using Lipofectamine 2000 (Invitrogen). 48 h after transfection, cells were selected by culturing with 20 μg/μl blastocidin containing medium for 7 days to collect stably integrated PSC cell lines.

### Co-immunoprecipitation and Western blot

Using the wild-type iPSCs, *Txnip* KO iPSCs, and the Flag-tagged Txnip expressing iPSCs, immunoprecipitation and western blot were done as previously described [16]. Glutathione S-transferase (GST) pull-down assay in GST-Txnip- and Oct4-overexpressed 293 T cells was performed as previously described [16, 17]. Anti-Txnip and anti-β-Actin were from Cell Signaling Technology (Danvers, Massachusetts). Anti-Oct4 (sc-5279) was acquired from Santa Cruz Biotechnology; Anti-Sox2 (MAB2018) was obtained from R&D System; anti-Nanog (ab14959), anti-H3K9ac (ab4441), anti-H3K4me3 (ab8580), anti-H3K27ac (ab4729), anti-H3 (ab1792), and anti-Hdac1 (ab7028) were purchased from Abcam; anti-Flag tag was purchased from (Sigma-Aldrich); anti-Pax6 (PRB-278P) was acquired from Covance; and anti-H3K27me3 (9733) was obtained from Millipore.

### Immunofluorescence and confocal microscopy

iPSCs were grown on coverslips for 2 days in LIF-containing media, induced to differentiate on coverslips by LIF withdrawal and RA induction for 3 days, or formed embryonic bodies (EBs) for 6 days. For EB immunostaining, EBs were harvested and fixed with 4% paraformaldehyde for 1 h at room temperature (RT). Fixed cells were then washed three times with PBS and further preserved in 30% sucrose at 4 °C, overnight. Sucrose-soaked EBs were then embedded in optimal cutting temperature (O.C.T) compound (Sakura Finetek). The frozen sections were obtained by cutting O.C.T blocks vertically into 10-um thickness using cryostat microtome. For immunofluorescence experiment, cells plated on 0.2% gelatin-coated coverslips were fixed with 4% paraformaldehyde for 15 min at RT and washed three times with PBS. The cells were permeabilized in 0.25% Triton X-100 and 3% BSA in PBS for 30 min at RT. Next, the cells were blocked with 0.05% Triton X-100 and 3% BSA in PBS for an hour at RT and were incubated overnight with each indicated primary antibodies at 4 °C. For in situ PLA assay, cells were fixed and permeabilized as described above. In situ interaction was performed using Duolink assay kit (Sigma) as previously described [17]. Antibody dilutions were 1:200 for anti-Oct4 (Santa Cruz Biotechnology, sc-5279), 1:200 for anti-Sox2 (R&D System, MAB2018), 1:200 for anti-Nanog (Abcam, ab14959), 1:200 for anti-Sox17 (R&D System, MAB1924), 1:200 for anti-Foxa2 (Millipore, 07-633), 1:200 for anti-Sma (Sigma-Aldrich, A2547), 1:200 for anti-Tuji (Covance, PRB-435P), 1:100 for anti-Nestin (Santa Cruz Biotechnology, sc-23927), 1:200 for anti-Pax6 (Covance, PRB-278P), and 1:200 for anti-H3K27ac (Abcam, ab4729). Secondary antibodies used were Alexa Fluor 488 and 546 (Invitrogen) and the nucleus was stained with DAPI. Confocal micro-images were obtained by a LSM800 confocal laser scanning microscope (Carl Zeiss).

### Chromatin immunoprecipitation (ChIP) assay

Chromatin immunoprecipitation (ChIP) assays were performed as described [18]. Briefly, iPSCs grown in high glucose (25 mM), low glucose (5 mM), and RA-induced differentiation conditions were cross-linked with 1% formaldehyde were lysed and sonicated. Lysates were diluted ten-fold in IP buffer and incubated overnight at 4 °C with appropriate amount of antibodies. Next day, the chromatin samples were incubated with protein A/G Plus agarose for 4 h at 4 °C, washed and further eluted. The eluted chromatin samples were then reverse cross-linked overnight at 65 °C. DNA was precipitated with ethanol and stored at − 20 °C till further use. The sequences of the ChIP-primers are listed in Table S2.

### Real-time qPCR

Total RNA was extracted from iPSCs with TRIzol reagent (Invitrogen). RNAs were extracted and synthesized into cDNAs, according to the manufacturer’s protocol (TOPscript™ RT DryMIX, Enzyomics). Quantitative real time-PCR for cDNAs was performed using the Fast SYBR™ Green Master Mix (ThermoFisher) on the 500 Fast Real-Time PCR system (Applied Biosystems), and normalized to *Gapdh*. For quantitating ChIP results, Fast SYBR™ Green Master Mix (ThermoFisher) was used and normalized to 1% input chromatin. Sequences of the primers for real-time PCR are listed in Table S2.

### RNA-seq data analysis

The extracted RNA samples were analyzed on an Agilent 2100 Bioanalyzer system (Agilent Biotechnologies). Only high-quality RNA samples (RNA integrity number ≥ 7.5) were used for preparing the samples for sequencing. cDNA libraries were prepared using NEBNext Ultra II Directional RNA-seq kit (New England Biolabs Inc.) according to manufacturer specifications. RNA-seq was performed on Illumina NovaSeq 6000 (Illumina) following the standard Illumina RNA-seq protocol with a read length of 2 × 100 bases. Reads of each sample were aligned to the mouse genome (NCBI build 38/mm10) using TopHat [19] with the default settings. Cufflinks [20] tools were used to quantify FPKM values and Edge R [21] tool were used to normalize genes defined from RefSeq transcripts. Unsupervised hierarchical clustering analysis of gene expression values were done by Cluster 3.0 [22] and the results were visualized with Java TreeView (http://jtreeview.sourceforge.net).

### Acetyl-CoA quantification

The total level of acetyl-CoA was determined by Acetyl CoA Assay Kit (Abcam, ab87546) following the manufacturer's instructions. Deproteinized total cell lysate of each sample was measured and acetyl-CoA concentration was normalized to the protein content of the corresponding samples. Data were collected from triplicate experiments.

### Lactate assay

The lactate levels were detected by using the L-Lactate Assay Kit (Abcam, ab65330) following the manufacturer's instructions. Briefly, cell lysates of each conditions were mixed with manufacturer-provided reaction mix and incubated for 30 min. The quantification was measured by luminescence (Molecular Devices). The lactate concentrations were calculated from the standard curve (0–10 nmol/ well) generated from the lactate standards according to the manufacturer’s instructions. Data were collected from triplicate experiments.

### Reporter gene assay

WT, *Txnip* KO iPSCs, *Scr*, and sh*Txnip* iPSCs were cultured and transfected with the reporter plasmids pOct4 (10x) TATA-luc using Lipofectamine 2000 (Invitrogen), as previously described [16]. Renilla luciferase activities were used to normalize transfection efficiencies. After 48 h, luciferase gene activity was measured using a microplate reader (Molecular Devices) following the manufacturer’s assay protocol.

### In Vivo teratoma formation assay

1 × 10^6^ WT and *Txnip* KO iPSCs were collected, mixed with matrigel (Corning, 354234) in 1:1 ratio, and injected subcutaneously into the BALB/c-nude mice (Jackson Laboratory). The mice were sacrificed after 5 weeks. Excised teratomas were fixed in 10% formalin solution and paraffin-embedded. The sections were evaluated by H&E staining.

### Mice

All animal experiments were performed in accordance with the guidelines of the Korea Research Institute of Bioscience and Biotechnology (KRIBB) Institutional Animal Care and Use Committee (Animal Welfare Assurance Number: KRIBB-AEC-22261).

## Results

### Txnip depletion promotes reprogramming to induced pluripotency

**Fig. 1:**
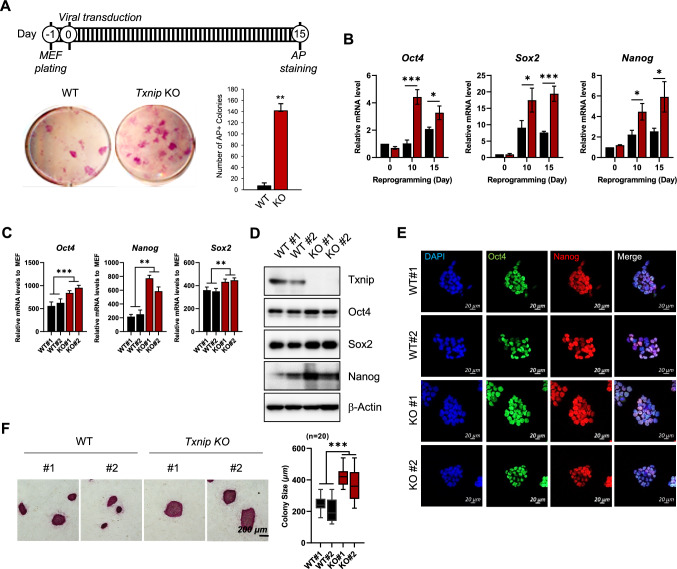
Txnip depletion facilitates generation of mouse iPSCs that continue to self-renew. **A** AP staining in WT and *Txnip* KO cells at reprogramming day 15. The number of AP positive colonies were counted and presented by graph. *Txnip* KO MEFs significantly induced a greater number of iPSC colonies compared to WT. (n = 3) Presented as means ± SEM. (**p ≤ 0.01) **B** The mRNA levels of *Oct4*, *Sox2*, and *Nanog* during iPSC induction in WT and *Txnip* KO MEFs. (n = 3) Presented as means ± SEM. (*p ≤ 0.05, ***p ≤ 0.001) **C**–**D** The mRNA and protein levels of *Oct4*, *Sox2*, and *Nanog* in WT and *Txnip* KO iPSCs. (n = 3) Presented as means ± SEM. (**p ≤ 0.01, ***p ≤ 0.001) Expression was detected by indicated antibodies; β-Actin was used as an internal control. **E** Representative immunofluorescence images of Oct4 and Nanog in WT and *Txnip* KO iPSCs. **F** AP staining in WT and *Txnip* KO iPSCs. *Txnip* KO iPSCs tend to grow to a larger colony size than WT iPSCs. (n = 3) Presented as means ± SEM. (***p ≤ 0.001)

To explore the role of Txnip in stem cell pluripotency, wild-type (WT) and *Txnip* knock-out (KO) mouse embryonic fibroblasts (MEFs) were delivered with competent sendai virus (SeV) that carry Yamanaka factors to reprogram MEFs into iPSCs, as previously reported [12, 13]. As expected, on day 10–15 post transduction, the cell colonies on the MEF culture dishes become large and compact, only consisting a fraction of iPSC colonies. Evaluated by alkaline phosphatase (AP) staining, WT and *Txnip* KO reprogrammed cells were identified whether they gained self-renewal activity which is one of iPSC characteristics. Interestingly, compared to WT MEF-derived iPSCs, *Txnip* KO MEF-derived iPSCs appeared to have greater number of AP positive colonies than WT MEF-derived iPSCs by nearly 15-fold (Fig. 1A). Moreover, during reprogramming process, the major pluripotency-associated genes, Oct4, Sox2, and Nanog, gained earlier and higher transcriptional induction in *Txnip* KO cells than WT cells (Fig. 1B). The reprogramming efficiency of *Txnip* KO was calculated to be ~ 1.696 (± 0.154) %, which was nearly 20-fold higher than the reprogramming efficiency of WT of ~ 0.084 (± 0.064) %.

We then established multiple monoclonal WT and *Txnip* KO iPSC lines. When each iPSC lines were cultured in serum and LIF-containing media conditions, *Txnip* KO iPSCs showed enhanced mRNA and protein levels of *Oct4, Sox2,* and *Nanog* (Fig. 1C and D), which were mainly located in the nucleus (Fig. 1E). In addition, both WT and *Txnip* KO iPSCs expressed compact AP positive colonies but differed in the average colony size (Fig. 1F), suggesting that *Txnip* KO iPSCs have enhanced cell proliferative characteristic.

Intrigued by relatively fast acquisition and enhancement of the pluripotency-associated factors in *Txnip* KO iPSCs, we tested whether Txnip activity could directly affect the properties of iPSCs. We first confirmed that PSCs including ESCs and iPSCs had higher mRNA and protein levels of Txnip (Fig. S1). Next, we introduced pLKO.1 lentiviral vector with small hairpin RNA (shRNA) of *Txnip* (sh*Txnip*) construct into WT iPSCs and assessed for change in iPSC characteristics. AP staining of control and sh*Txnip* iPSCs displayed no change in self-renewal ability (Fig. S2A). In addition, mRNA and protein expression of the pluripotency-associated genes were still well expressed even though Txnip expression level was reduced by half (Fig. S2B–D), suggesting that down-regulation of Txnip does not influence the PSC status in the presence of serum and LIF. Altogether, these results demonstrated that Txnip deficiency accelerates induced pluripotency, thereby being more efficient in iPSC reprogramming, but has no further effect on maintaining the undifferentiated state of iPSCs in the presence of serum and LIF.

### Loss of Txnip impairs initial multi-lineage differentiation of PSCs

Txnip is involved in many cellular processes, including the proliferation, differentiation, apoptosis, metabolism, and inflammation [23]. Indeed, Txnip is induced during erythroid differentiation [24] and its knockdown suppresses keratinocyte differentiation [25], although *Txnip* KO mice undergo normal development in vivo. Moreover, Txnip is recently reported as one of the constitutively active genes induced in human iPSCs and highly upregulated in the differentiated states independent of upstream p53 activation [26]. Hence, we seek to examine whether Txnip is related to in vitro differentiation of PSCs.

**Fig. 2:**
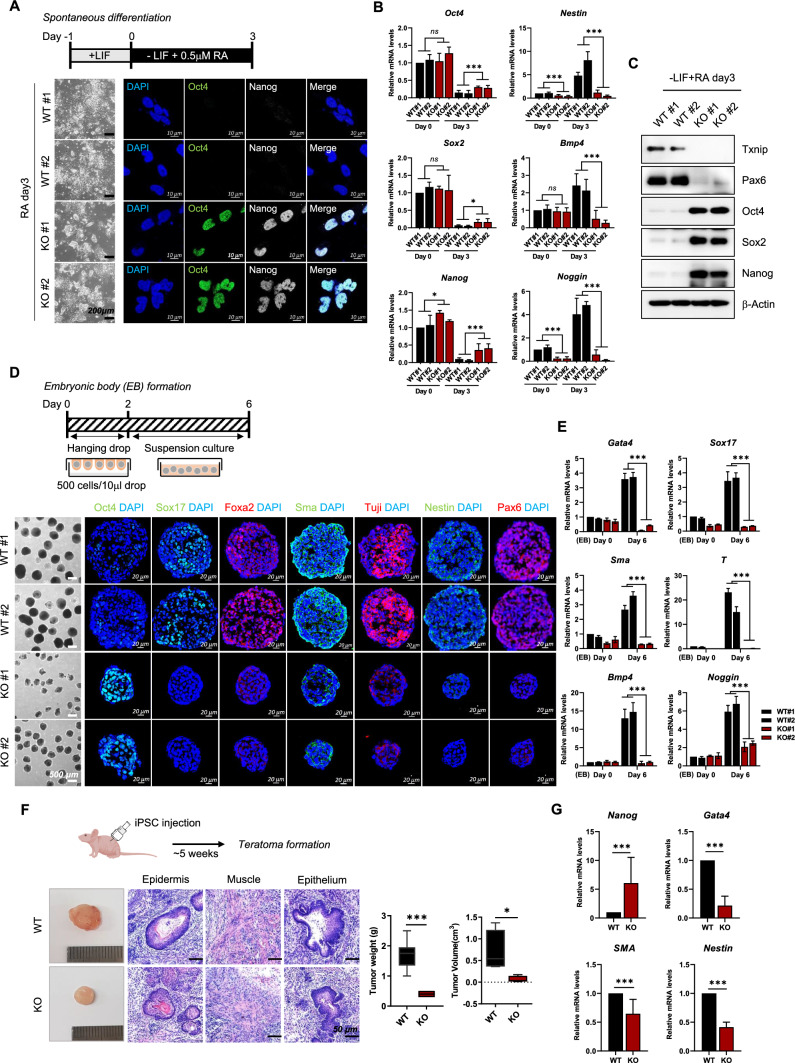
Txnip is required for exit from the pluripotent state in vitro and in vivo. **A** Representative morphology and immunofluorescence images of spontaneous differentiation of WT and *Txnip* KO iPSCs. Spontaneous differentiation of iPSCs was induced by LIF withdrawal from PSC medium and addition of RA. *Txnip* KO iPSCs showing more delayed differentiation rate than WT iPSCs was presented by Oct4 and Nanog expressions in RA-induced differentiated cells. **B**–**C** The mRNA and protein levels of *Oct4*, *Sox2*, and *Nanog*, and Ectoderm lineage genes in WT and *Txnip* KO iPSCs upon RA-induced differentiation for 3 days. Expression was detected by indicated antibodies; β-Actin was used as an internal control. (n = 3) Presented as means ± SEM. (*p ≤ 0.05, **p ≤ 0.01) **D** Representative morphology and immunofluorescence images of embryoid body (EB) formation of WT and *Txnip* KO iPSCs for 6 days. iPSCs were differentiated as EBs by hanging drop method (500 cells/10 µl drop) in the absence of LIF for 2 days followed by 4-day suspension culture. *Txnip* KO EBs showed rather smaller size and presented reduced expression of three germ layer markers than that of WT. **E** The mRNA levels of three germ layer marker genes in WT and *Txnip* KO iPSCs upon EB differentiation for 6 days. (n = 3) Presented as means ± SEM. (***p ≤ 0.001) **F** Histological analysis of teratomas from WT and *Txnip* KO iPSCs by H&E staining. iPSCs were injected subcutaneously into the BALB/c-nude mice. WT and *Txnip* KO-derived teratomas were compared by tumor weight and volume. (n = 3) Presented as means ± SEM. (*p ≤ 0.05, ***p ≤ 0.001) **G** The mRNA levels of *Nanog*, *Gata4*, *Sma*, and *Nestin* in WT and *Txnip* KO-derived teratomas. (n = 3) Presented as means ± SEM. (***p ≤ 0.001)

WT and *Txnip* KO iPSCs were first differentiated spontaneously by LIF withdrawal and retinoic acid (RA) addition for 3 days (Fig. 2A). WT iPSCs were stably differentiated without showing any iPSC colonies and having negative Oct4, Sox2, and Nanog expressions (Fig. 2A–C). However, RA-induced *Txnip* KO iPSCs displayed somewhat different morphology from that of WT iPSCs, by having more leftover undifferentiated iPSC colonies (Fig. 2A) and still expressing Oct4, Sox2, and Nanog (Fig. 2A–C). Consequently, ectodermal lineage markers, Nestin, Bmp4, Noggin, and Pax6, were induced in RA-differentiated WT cells but still suppressed its expression levels in RA-differentiated *Txnip* KO cells (Fig. 2B and C), indicating that differentiation of *Txnip* KO iPSCs may be inhibited or delayed.

We next examined the differentiation potential of *Txnip* KO iPSCs by using an in vitro embryoid body (EB) foramtion method. Even after 6 days of EB differentiation, *Txnip* KO EBs still retained relative Oct4 expression compared to WT EBs (Fig. 2D). Moreover, *Txnip* KO EBs displayed rather smaller size of EBs compared to WT EBs, giving its possibilities in defect of in vitro three germ layer differentiation. By using immunostaining methods, representative markers of three germ layers which are Sox17 and Foxa2 for endoderm, Sma for mesoderm, and Tuji, Nestin, and Pax6 for ectoderm were assessed. Compared to WT EBs which expressed all three germ layer markers, *Txnip* KO EBs showed reduce expression of all differentiation-associated markers (Fig. 2D). Differentiation bias of *Txnip* KO iPSCs were also investigated using qRT-PCR and found out that markers of the three germ layers were reduced in *Txnip* KO iPSCs compared with WT iPSCs, suggesting that Txnip loss retards spontaneous differentiation of iPSCs (Fig. 2E).

To further explore this phenomenon in vivo, WT and *Txnip* KO iPSCs were injected subcutaneously into nude mouse to generate teratomas (Fig. 2F). Although, Txnip has tumor suppressive, metastasis inhibitory, and proapoptotic function [27], *Txnip* KO iPSCs consistently generated smaller teratomas compared to WT iPSCs-derived teratomas (Fig. 2F). Histological analysis confirmed that WT iPSCs and *Txnip* KO iPSCs generated teratomas that included, epidermis (ectoderm lineage), muscle (mesoderm lineage), and epithelium (ectoderm lineage), but *Txnip* KO iPSCs-derived teratomas having areas of focally immature cells, existent *Nanog* and insufficient differentiation-associated markers, consistent with reduced differentiation (Fig. 2F and G). These data indicate that iPSCs lacking Txnip impede spontaneous differentiation in vitro and in vivo that may apply to cell-fate decisions.

### Txnip is essential for global transcription changes upon PSC differentiation

To further investigate biological effect of Txnip during iPSC generation and differentiation, we conducted RNA-sequencing method in WT and *Txnip* KO MEFs, iPSCs, and iPSC-derived EBs. All RNA-seq data was filtered 0.5 counts per million, or CPM and generated a bar plot of total read counts per library which shows small variation in library sizes (Fig. S3A). Data was log transformed (Fig. S3B) and applied for differentially expressed gene (DEG) analysis (Figs. S3E and 3F). Principal component analysis (PCA) plot showed clear difference between WT and *Txnip* KO samples (Fig. S3C) and scatter plots for sample replicates confirmed a high degree of correlation (Fig. S3D). Gene ontology (GO) analysis in WT and *Txnip* KO iPSCs DEGs presented *Txnip* KO downregulates biological processes related to various organ morphogenesis and development and upregulates genes related to LIF responses (Fig. S3G). Moreover, WT and *Txnip* KO EBs DEGs were positively correlated with cellular metabolic processes (Fig. S3H).

**Fig. 3:**
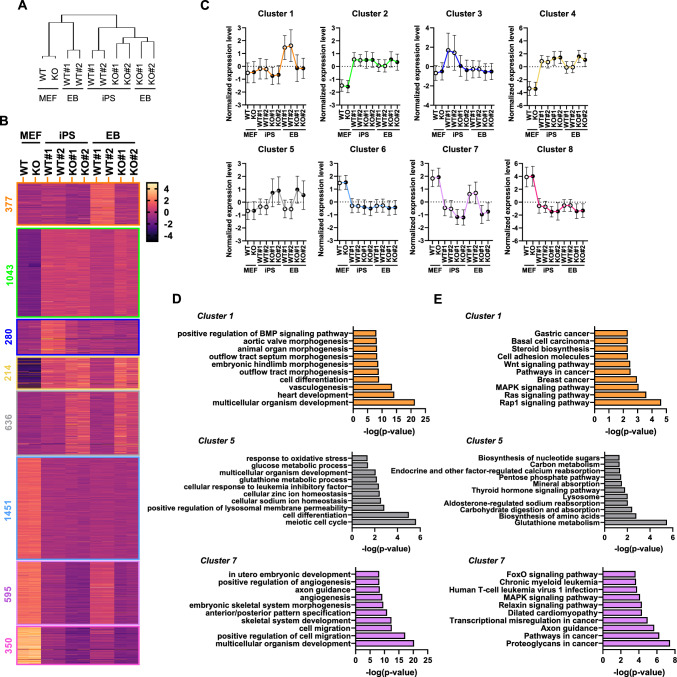
Txnip deficiency results in global transcription changes upon spontaneous differentiation. **A** A hierarchical clustering tree of WT MEFs, *Txnip* KO MEFs, WT iPSCs, *Txnip* KO iPSCs, WT EBs, and *Txnip* KO EBs. **B** Eight expression patterns are shown on the left. Heatmap drawn based on k-clustering results shows the relative expression levels of each transcript (rows) in each sample (column). **C** The average of log2-normalized data corresponding genes in each pattern. **D**–**E** Gene ontology (GO) enrichment analysis and KEGG pathway for differentially expressed genes in cluster 1, 5, and 7, representatively. All differentially expressed genes were subjected to GO analysis—the top enriched terms are shown

Through hierarchical clustering analysis, WT EBs were closely clustered with WT and *Txnip* KO MEFs, while *Txnip* KO EBs were clustered with both WT and *Txnip* KO iPSCs (Fig. 3A). This indicated that *Txnip* KO EBs exhibit gene profiles similar to the undifferentiated iPSC state, supporting the earlier findings that *Txnip* KO EBs display a differentiation defect (Fig. 2D and E). To further explore the functional diversity of DEGs, we performed a K-means clustering analysis and grouped the 14,336 DEGs into 8 clusters (Fig. 3B and Table S1). Interestingly, genes in cluster 1 increased during EB differentiation of WT iPSCs, but their expression remained reduced in *Txnip* KO EBs. This gene cluster is mainly involved in “multicellular organ development”, “vasculogenesis”, “cell differentiation”, and “various organ morphogenesis” (Fig. 3D). Also, related to pathways represented MAPK pathway, WNT signaling pathway, and cancer-related pathways (Fig. 3E).

Similarly, *Txnip* KO EBs showed highly delayed or reduced gene expressions than WT EBs in cluster 7 and 8 (Fig. 3C), including gene ontologies of “angiogenesis”, “cell migration”, and “multicellular organism development” (Fig. 3D) and pathways of “FoxO signaling”, “MAPK signaling”, and “cancer” (Fig. 3E). These were also correlated to the results that *Txnip* KO teratomas displayed defect in formation and in vivo differentiation (Fig. 2F and G). We confirmed that *Txnip* KO teratomas led to a significant reduction in various angiogenesis-related genes, including those in cluster 7 *(Ets1* and *Flt1)* and cluster 8 *(Thbs1* and *Nrp1)* (Table S1 and Fig. S4). This suggests that the reduction may be due to defect in the proangiogenic role during teratoma formation of *Txnip* KO iPSCs, considering that tumor-derived angiogenic factors are crucial in forming neovascularization in solid tumors [28]. Overall, these results demonstrate that Txnip may be required to occur global transcription changes upon stem cell differentiation process.

### Txnip deficiency drives the metabolic shift toward glycolytic energy production

**Fig. 4:**
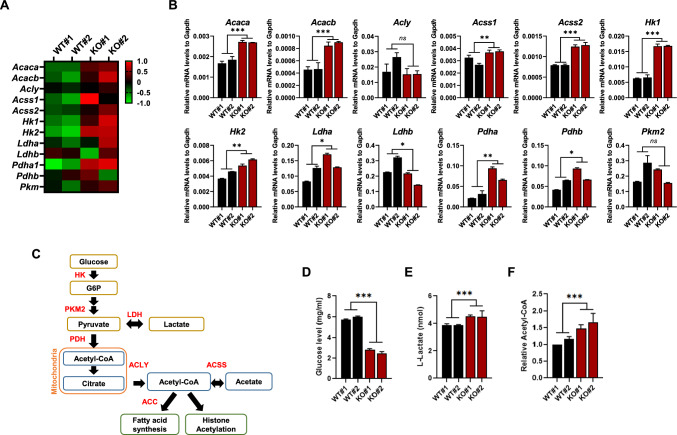
*Txnip* KO iPSCs show induced glucose uptake through upregulation of glycolytic enzymes. **A** A heatmap of differentially expressed genes between WT and *Txnip* KO MEFs and iPSCs involved in glucose metabolism. **B** The differentially expressed mRNA levels of metabolic genes in WT and *Txnip* KO iPSCs. Real-time qPCR of *Acaca/b, Acly, Acss1/2, HK1/2, Ldha/b, Pdha/b,* and *Pkm2* in WT and *Txnip* KO iPSCs. (n = 3) Presented as means ± SEM. (*p ≤ 0.05, **p ≤ 0.01, ***p ≤ 0.001, and *ns,* not significant.) **C** A schematic diagram showing which gene corresponds to which metabolic stage. **D**–**F** Txnip elevates glucose metabolism and increases the production of by-products; *Txnip* KO iPSCs showed relatively lower glucose level in the culture media (**D**), higher intracellular L-Lactate levels (**E**), and relatively higher level of intracellular acetyl-coA (**F**). (n = 3) Presented as means ± SEM. (***p ≤ 0.001)

The metabolic transition from Oxphos to glycolysis is essential for efficient iPSC reprogramming and iPSCs rely on glycolysis to provide energy and substrates to maintain pluripotency [3, 6]. Txnip is also known as an important regulator of glucose and lipid metabolism [29], suggesting that Txnip loss may modulate metabolic changes associated the entry and exit of pluripotency. Interestingly, we found that the expression of genes involved in cellular metabolic processes, such as glycolytic enzyme hexokinase 2 (Hk2), previously reported to be upregulated by Txnip knockdown [30], was consistently more activated in both *Txnip* KO- iPSCs and EBs than in WT- iPSCs and EBs, respectively, but not different between WT and *Txnip* KO MEFs, as shown in cluster 5 (Fig. 3D, E and Table S1). Therefore, we next investigated from RNA-seq data (Fig. 4A) and validated by qRT-PCR (Fig. 4B) whether metabolic genes involved in glycolysis processes that generate acetyl-coA, acetate and fatty acid (Fig. 4C) in iPSCs show changes in expression upon Txnip loss. Quantitative gene expression analysis showed higher expression of hexokinase (*Hk1* and *Hk2*), pyruvate dehydrogenase (*Pdha* and *Pdhb*), acetyl-coenzyme A synthetase (*Accs1* and *Accs2*), and acetyl-coenzyme A carboxylase (*Acaca* and *Acacb*) in *Txnip* KO iPSCs than WT iPSCs (Fig. 4B).

Increased rate of glycolysis in *Txnip* KO iPSCs was validated by decreased glucose level in *Txnip* KO iPSC medium which means higher glucose uptake (Fig. 4D), as previously reported in MEFs [11]. Increased glucose uptake in *Txnip* KO iPSCs was still maintained in condition of low glucose (5 mM) medium (Fig. S5A). Upregulated glycolysis level also increased lactate production in *Txnip* KO iPSCs in both high glucose (25 mM) (Fig. 4E) and low glucose (5 mM) conditions (Fig. S5B). In relation to enhanced lactate production in *Txnip* KO iPSCs, induced lactate dehydrogenase (*Ldha*) and reduced *Ldhb* in *Txnip* KO iPSCs were detected (Fig. 4B). Moreover, increased in glycolysis rate lead to increase in acetyl-coA levels which showed 1.5- to 2-fold induction of acetyl-coA concentration in *Txnip* KO iPSCs compared to WT iPSCs (Fig. 4F). These results demonstrate that Txnip deficiency may drive induced pluripotency, but hinder differentiation by retaining metabolic enzymes essential for stemness maintenance.

### Txnip regulates histone acetylation levels through changes in metabolic profile

**Fig. 5:**
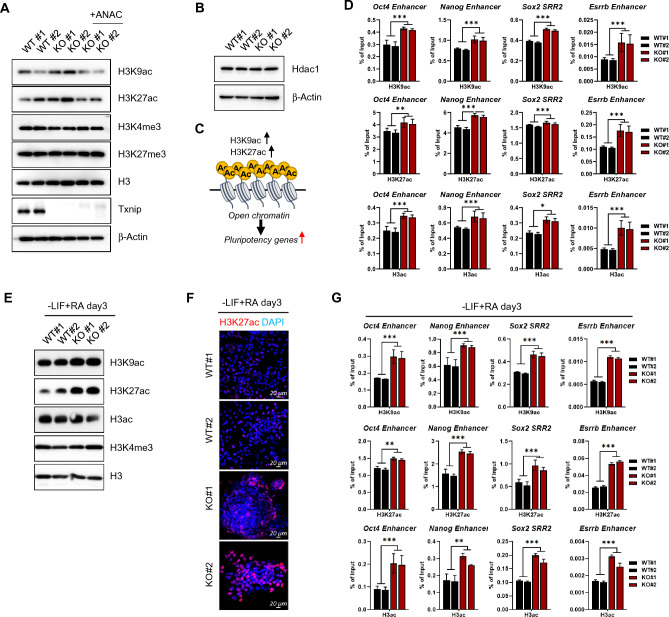
*Txnip* KO iPSCs are enriched for histone H3 acetylation at pluripotency-associated genes. **A** The protein levels of histone H3 acetylation and methylation were assessed by using indicated antibodies. *Txnip* KO iPSCs showed elevated histone acetylation (H3K9ac and H3K27ac) levels and treatment of anacardic acid (ANAC), a histone acetyltransferase (HAT) inhibitor, regained its normal expression. **B** The unchanged protein expression level of Hdac1 showed that enriched histone acetylation is not related to change in Hdac1 levels due to Txnip loss. **C** A simplified schematic of histone acetylation (H3K9ac and H3K27ac) as an active mark of open chromatin in the pluripotent state. **D** ChIP-qPCR analysis of H3K9ac, H3K27ac, and H3ac on active PSC gene such as *Oct4, Nanog, Sox2*, and *Esrrb* regions in WT and *Txnip* KO iPSCs. (n = 3) Presented as means ± SEM. (*p ≤ 0.05, **p ≤ 0.01, ***p ≤ 0.001) *Txnip* KO iPSCs showed higher enrichment of histone acetylation than WT. **E** The protein levels of histone acetylation in RA-induced differentiation state. Increase in global histone acetylation levels were presented in RA-induced *Txnip* KO differentiated samples compared to that of WT. **F** Representative immunofluorescence images of H3K27ac levels in RA-induced differentiation of WT and *Txnip* KO iPSCs for 3 days. **G** ChIP-qPCR analysis of H3K9ac, H3K27ac, and H3ac on active PSC gene such as *Oct4, Nanog, Sox2*, and *Esrrb* regions in RA-induced differentiated WT and *Txnip* KO cells. (n = 3) Presented as means ± SEM. (*p ≤ 0.05, **p ≤ 0.01, ***p ≤ 0.001)

Acetyl-coA is a well-known substrate of acetylation, leading to a hypothesis that Txnip loss might affect the histone acetylation state of iPSCs. We first detected global H3K9ac and H3K27ac levels by western blot, and showed that *Txnip* KO iPSC samples have enhanced H3K9ac and H3K27ac levels compared to WT iPSCs (Fig. 5A). As expected, histone methylation expressions were unaffected by Txnip (Fig. 5A). Enhanced histone acetylation levels in *Txnip* KO iPSCs were reduced by the histone acetylase inhibitor, anacardic acid (ANAC) (Fig. 5A). Also, The lack of change in histone deacetylase 1 (Hdac1) expression indicated that the increase in histone acetylation in *Txnip* KO iPSCs was not associated with Hdac1 (Fig. 5B).

Next, we speculated knock-out effect of Txnip on histone acetylation occupancy on active pluripotency-associated gene regions (Fig. 5C). Enhanced H3K9ac, H3K27ac, and H3ac occupancy was detected in Oct4, Nanog, Sox2, and Esrrb enhancer regions validated by chromatin immunoprecipitation (ChIP)-qPCR experiments (Fig. 5C). Since *Txnip* KO iPSCs were able to maintain the increased histone acetylation levels under low glucose conditions compared to WT iPSCs (Fig. S5C), the extent of histone acetylation increase in *Txnip* KO iPSCs on active gene regions changes to a greater extent (Fig. S5D). This further led to upregulated Oct4 occupancy in Oct4, Nanog, Sox2, and Esrrb enhancer regions (Fig. S5E) and maintained Oct4, Sox2, and Nanog expression in low glucose conditions (Fig. S5F). These results suggest that sustained histone acetylation levels by upregulated glycolytic metabolism in *Txnip* KO iPSCs may enable pluripotent stem cells to be maintained even in a insufficiently glucose environment.

We further investigated whether induced histone acetylation levels were maintained throughout PSC differentiation. WT iPSCs and *Txnip* KO iPSCs were induced to differentiate by LIF withdrawal and RA treatment for 3 days. H3K9ac, H3K27ac, and H3ac levels were still increased in RA-differentiated *Txnip* KO cells compared to RA-differentiated WT cells while H3K4me3 levels were left unchanged (Fig. 5D). Increased H3K27ac levels were also detected in RA-differentiated *Txnip* KO cells by immunostaining (Fig. 5E). By ChIP-qPCR experiments, the occupancies of H3K9ac, H3K27ac, and H3ac on active stemness gene regions were more increased in RA-differentiated *Txnip* KO cells than in wild-type (Fig. 5F). The above results also support that the expression of pluripotency-related genes in *Txnip* KO iPSCs is sustained even during the onset of differentiation.

### Txnip represses Oct4-dependent gene expression by acting as a transcriptional corepressor

**Fig. 6:**
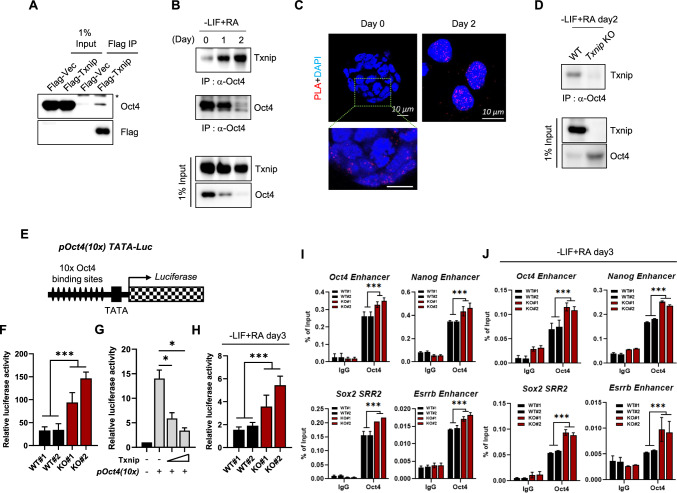
Txnip directly interacts with Oct4 and inhibits its activity during differentiation. **A** Whole-cell lysates from WT iPSCs expressing Flag-Txnip were immunoprecipitated with α-Flag antibody. Bound Oct4 proteins were detected with α-Oct4 antibody. Asterisk indicates IgG heavy chain. **B** Co-immunoprecipitation (co-IP) of Txnip and Oct4 proteins by RA-induced differentiation of WT iPSCs. Cell lysates were immunoprecipitated with α-Oct4 antibody and probed with α-Txnip or α-Oct4 antibody **C** In situ proximity ligation assay (PLA) images in WT iPSCs and RA-induced differentiated cells. PLA detected the physical interaction of Txnip and Oct4 in situ (at distances < 40 nm) at endogenous protein levels. **D** Co-IP of Txnip and Oct4 proteins in WT iPSCs and *Txnip* KO iPSCs by RA-induced differentiation. Cell lysates were immunoprecipitated with α-Oct4 antibody and probed with α-Txnip antibody. **E** Schematic representation of pOct4(10x) TATA-luc reporter plasmid. **F** pOct4(10x) reporter activity of WT and *Txnip* KO iPSCs. WT and Txnip KO iPSCs were transiently transfected with pOct4(10x) TATA-luc reporter plasmid. Renilla luciferase activities were used to normalize transfection efficiencies. Luciferase activity was measured 48 h after transfection. (n = 3) Presented as means ± SEM. (***p ≤ 0.001) **G** Reduction of pOct4(10x) reporter activity by overexpression of *Txnip* into WT iPSCs. (n = 3) Presented as means ± SEM. (*p ≤ 0.05) **H** RA-induced WT and *Txnip* KO differentiated cells were transiently transfected with pOct4(10x) reporter plasmid. (n = 3) Presented as means ± SEM. (***p ≤ 0.001) **I** ChIP-qPCR analysis of Oct4 on active pluripotency-associated Oct4 target gene such as *Oct4, Nanog, Sox2,* and *Esrrb* regions in WT and *Txnip* KO iPSCs. (n = 3) Presented as means ± SEM. (***p ≤ 0.001) *Txnip* KO iPSCs showed higher Oct4 enrichment than WT. **J** ChIP-qPCR analysis of Oct4 on active gene regions in RA-induced differentiated WT and *Txnip* KO cells. (n = 3) Presented as means ± SEM. (***p ≤ 0.001)

Txnip is well-known to inhibit the biological functions of binding proteins [17, 31]. Typically, Txnip binds to Trx and inhibits its activity [8]. Previous studies in PSCs reported that the molecular circuitry governing pluripotency hinges upon Oct4 [32–34], Thus, we hypothesized that Txnip not only indirectly regulates Oct4 expression through histone acetylation, but also participates in Oct4 inhibition in early differentiation by directly binding to Oct4. We first generated stably expressed Flag tagged Txnip-iPSCs (Fig. S6A and B) and verified interaction of Txnip with Oct4 by co-immunoprecipitation (co-IP) (Fig. 6A). Glutathione S-transferase (GST) pull-down assay also confirmed the interaction of Txnip and Oct4 using GST-Txnip- and Oct4-overexpressed 293 T cells (Fig. S7A).

Next, to investigate whether spontaneous differentiation affects their interaction, we induced early differentiation of WT and *Txnip* KO iPSCs by LIF withdrawal and RA induction (Fig. 6B–D). By RA-induced differentiation, Oct4 was quickly decreased, thereby Oct4 self-association was also reduced in WT iPSCs (Fig. 6B). Interestingly, Txnip expression was maintained during early differentiation, but that its binding affinity to Oct4 is increased continuously, although Oct4 was strongly suppressed upon RA-induced differentiation (Fig. 6B and D). In addition, In situ proximity ligation assay (PLA) confirmed that the physical interaction of Txnip and Oct4 in situ (at distances < 40 nm) at endogenous protein levels was increased by LIF withdrawal and RA induction (Fig. 6C). As previously described, Txnip deficiency resulted in maintain residual Oct4 expression (Fig. 6D).

Oct4 transactivation level was tested by transiently transfecting pOct4(10x) TATA luciferase vector harboring 10 copies of Oct4 recognition sites [16] into WT and *Txnip* KO iPSCs. Significantly, higher luciferase activity was detected in *Txnip* KO iPSCs (Fig. 6F). Oct4 transcriptional activity was also measured when increment dose of Txnip was expressed in WT iPSCs (Fig. 6G) and 293 T cells (Fig. S7B). With addition of Txnip expression, the transcriptional activity of Oct4 decreased proportionally (Figs. 6G and S7B). Consistent with the sustained expression of Oct4 despite transitioning into differentiation, the increased Oct4 activity in *Txnip* KO cells were also maintained under RA-differentiated conditions (Fig. 6H).

In light of the significant differences of Oct4 activity revealed above, we confirmed the regulation of pluripotency-associated Oct4 targets by Txnip loss. As expected, increase of Oct4 expression in *Txnip* KO iPSCs by direct and indirect regulation led to Oct4 occupancy in active PSC gene regions (Fig. 6I). Moreover, even in differentiating conditions, *Txnip* KO iPSCs maintained Oct4 expression levels and subsequently its occupancy on Oct4 target genes (Fig. 6J) which may result in delay in pluripotency exit. Altogether, these results indicate that Txnip may play a critical role in faithful differentiation for PSC fate conversion by controlling histone acetylations and Oct4 activity for pluripotency.

## Discussion

**Fig. 7:**
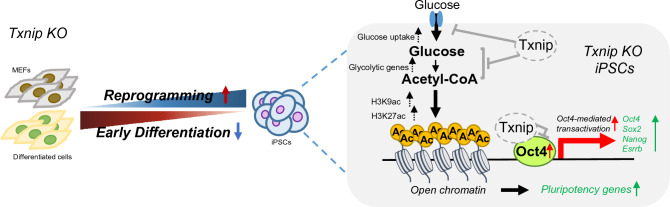
A cartoon illustrating the role of Txnip in switching the fate of pluripotent stem cells. Control of Txnip expression is crucial for cell fate transitions by modulating the entry and exit of pluripotency. *Txnip* KO indirectly regulates pluripotency-associated genes including *Oct4, Sox2, Nanog,* and *Esrrb* by regulating glucose-mediated metabolism through increasing expressions of multiple metabolic genes, and modulating histone H3 acetylation (H3K9ac and H3K27ac) levels, thereby adjusting the chromatin feature in pluripotent state. Moreover, Txnip directly interacts with Oct4 and inhibits its transcriptional activity, suggesting that Txnip expression at the onset of differentiation is important for Oct4 deregulation

Previous studies have shown that Txnip is involved in the regulation of proliferation, differentiation, apoptosis, autophagy, and inflammation in various cell types, such as beta, liver, peripheral, immune, and cancer cells [11, 17, 23]. In this study, for the first time, we revealed the novel functions of Txnip in PSCs, in induced pluripotency and differentiation by driving glucose-mediated histone acetylation and regulating the activity of Oct4 (Fig. 7).

*Txnip* KO MEFs successfully reprogrammed to pluripotency and appeared to have a greater number of AP positive iPS colonies compared to WT MEF-reprogrammed cells. Consistent with this phenomenon, *Txnip* KO cells highly activated pluripotency-associated genes such as *Oct4*, *Sox2*, and *Nanog* earlier than WT during the reprogramming process. As previously reported, stage-specific metabolic reprogramming is crucial for the efficient transition of cell fate from somatic cells to iPSCs and occurs before the induction of pluripotency markers [35]. Metabolic changes are closely linked to epigenetic remodeling and subsequent transcriptome changes. The observed enriched glycolysis-related genes in *Txnip* KO iPSCs implicates that Txnip may act as a metabolic sensor for changes in the cell environment and facilitate the glycolytic transcriptional response during reprogramming.

Although the stem cell phenotypes in *Txnip* KO iPSCs were similar to WT, importantly, we observed that *Txnip* KO iPSCs tend to differentiate poorly and maintain the expression of stemness factors in the process of switching to spontaneous differentiation in vitro and in vivo. This defect in differentiation was also demonstrated by global RNA-seq analysis, which revealed distinct profiles that segregated the WT and *Txnip* KO phenotypes with respect to pluripotency, differentiation, migration, angiogenesis, and metabolism gene expression by GO and KEGG analysis. Our data indicate that Txnip activity at the onset of early differentiation may be crucial for cell-fate transition, regardless of terminal lineage commitment. The detailed molecular mechanisms underlying differentiation towards specialized cells in *Txnip* KO still expect further investigation.

In contrast to the somatic reprogramming process, the forced transition from glycolysis to Oxphos is accompanied by a loss of PSC properties and increased differentiation changes [3]. As mentioned above, loss of Txnip resulted in an increase in glycolysis and a concomitant increase in histone acetylation levels on pluripotency genes, thereby suppressing the differentiation of PSCs. Indeed, glycolytic-enzymes were upregulated in *Txnip* KO iPSCs and EBs, leading to cellular metabolic changes such as increased glucose consumption rate and upregulated acetyl-coA production, which are all cellular signatures of glycolytic flux induction. In addition, when exposed to insufficient glucose conditioned media, normally pluripotency genes of WT iPSCs rapidly adapt to the environment and downregulate their expression. However, pluripotency genes of *Txnip* KO iPSCs still maintained their expression under low glucose conditions because the loss of Txnip continuously activate glycolysis and enhance histone acetylation levels on pluripotency genes. Therefore, highlighting the important role of Txnip, which may be essential for adapting to cellular environmental cues.

Core pluripotency factors Oct4, Sox2, and Nanog are closely related to the glycolysis in the reprogramming process and maintenance of pluripotency. In particular, Oct4 can directly regulate the key enzymes of glycolysis such as *Hk2* and *Pgk1* [34], which were also identified as constitutively active expressed genes upon Txnip loss (Table S1). In addition to the indirect regulation of pluripotency genes by Txnip through histone acetylation, Txnip is able to interact with Oct4 and repress its transcriptional activity of known Oct4 targets. Therefore, our results suggest that Txnip possibly acts as a transcriptional corepressor similar to other cell types [31]; direct regulation of Txnip on Oct4 controls its genome occupancy on Oct4 target genes, which may further lead to cell fate changes in response to oxidative stress during the differentiation process. Further studies are needed to elucidate the missing link between the Txnip-Oct4 complex and glycolytic genes, as well as its synergistic effects with core stemness factors on iPSC reprogramming and differentiation.

In conclusion, our work in *Txnip* KO pluripotent and differentiated cells define a critical role of Txnip in regulating metabolic properties and mediating histone acetylation for chromatin accessibility, in which Txnip act as a corepressor and interacts with a master pluripotency factor Oct4 for cell fate decision in differentiating PSCs.

### Supplementary Information

Below is the link to the electronic supplementary material.

Supplementary file1 (DOCX 1938 KB)

Supplementary file2 (XLSX 547 KB)

## Data Availability

The datasets generated and/or analyzed in the current study are available on reasonable request. The total RNA-seq data are available in the Korean Nucleotide Archive (KoNA, https://kobic.re.kr/kona) public database with the accession number KAP230692.
