# Supplementary material for: Txnip regulates the Oct4-mediated pluripotency circuitry via metabolic changes upon differentiation
Source: Cell Mol Life Sci. 2024 Mar 15;81(1):142. doi: 10.1007/s00018-024-05161-y (PMC10940461; doi:10.1007/s00018-024-05161-y)
Supplement: Supplementary file 1 — Supplementary file1 (DOCX 1938 KB) [file 18_2024_5161_MOESM1_ESM.docx]

**Supplementary Information**

**Supplementary Figures**

**Supplementary Figure 1.** Txnip is expressed in pluripotent stem cells.

**Supplementary Figure 2.** Knockdown of *Txnip* has no effect on self-renewal ability of iPSCs.

**Supplementary Figure 3.** Pre-process diagnostic plots for read count data.

**Supplementary Figure 4.** Genes related to defect in *Txnip* KO teratoma formation.

**Supplementary Figure 5.** Pluripotency is maintained through low glucose conditions in *Txnip* KO iPSCs.

**Supplementary Figure 6.** Generation of Txnip overexpressed iPSCs.

**Supplementary Figure 7.** Txnip directly interacts with Oct4 and inhibits its activity in 293T cells.

**Supplementary Tables**

**Supplementary Table 1.** Differentially expressed genes grouped into eight clusters. (an Excel file)

**Supplementary Table 2.** Primers for RT-qPCR and ChIP-qPCR used in this study.

**
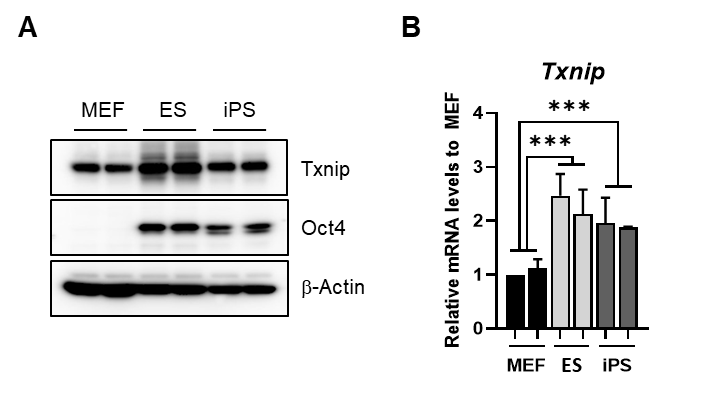
**

**Supplementary Fig. 1** Txnip is expressed in pluripotent stem cells.

**(A)** The protein levels of Txnip and Oct4 in MEF, J1 mouse ES, and WT iPS cells in serum and LIF conditions. Expression was detected by indicated antibodies; β-Actin was used as an internal control.

**(B)** The mRNA level of *Txnip* in MEF, J1 mouse ES, and WT iPS cells in serum and LIF conditions. (n=3) Presented as means ± SEM. (*** *p* ≤ 0.001)

**
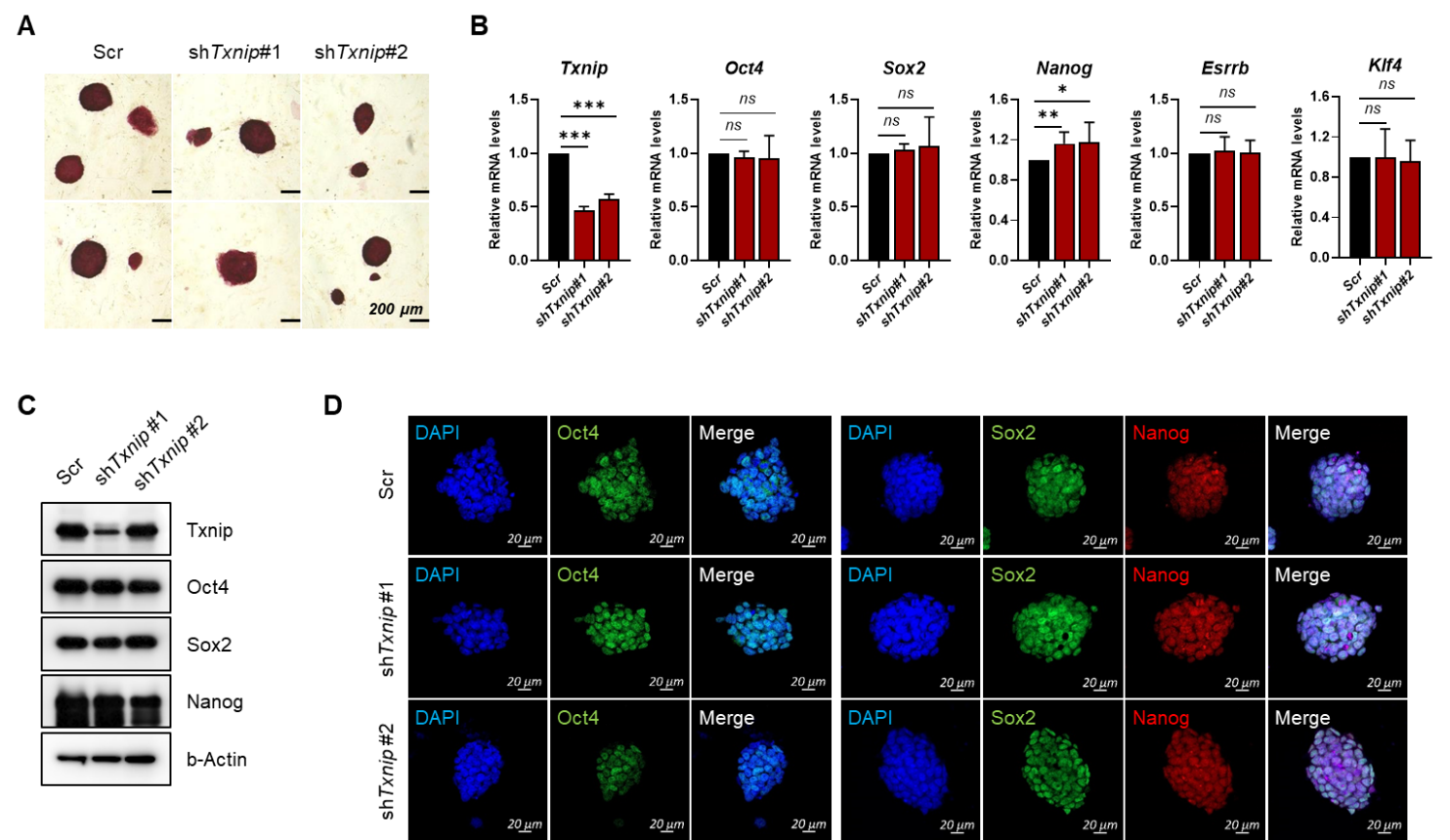
**

**Supplementary Fig. 2** Knockdown of *Txnip* has no effect on self-renewal ability of iPSCs.

**(A)** Self-renewal assay and alkaline phosphatase (AP) staining in scrambled and shRNA-introduced *Txnip* knockdown iPSCs.

**(B-C)** The mRNA and protein expression levels of pluripotency genes scrambled and sh*Txnip* iPSCs. Expression was detected by indicated antibodies; β-Actin was used as an internal control. (n=3) Presented as means ± SEM. (*ns*; not significant, * *p* ≤ 0.05, ** *p* ≤ 0.01, *** *p* ≤ 0.001)

**(D)** Representative immunofluorescence images of Oct4, Sox2, and Nanog in scrambled and sh*Txnip* iPSCs in serum/LIF conditions.

**
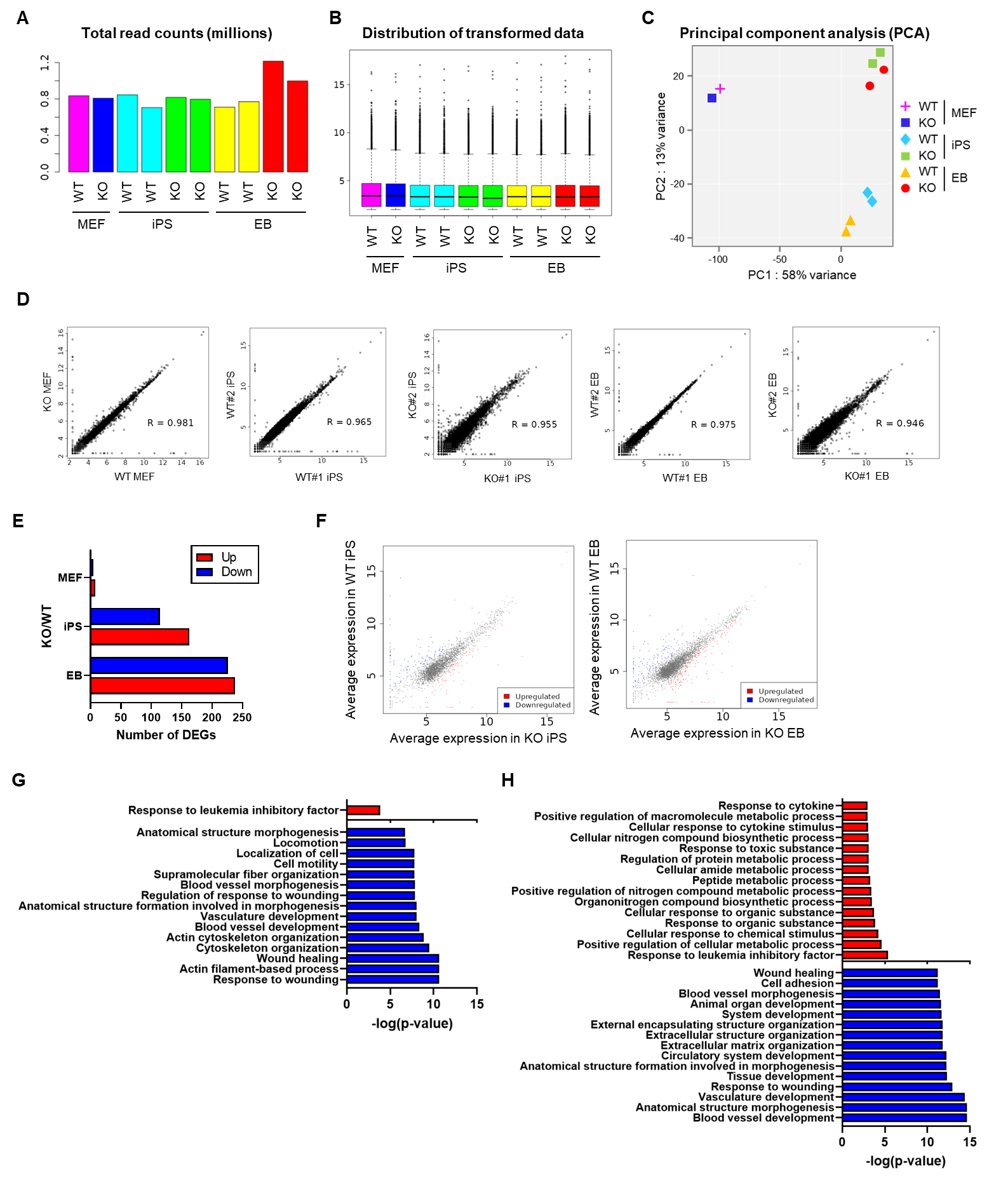
**

**Supplementary Fig. 3** Pre-process diagnostic plots for read count data.

**(A-D)** Diagnostic plots for read-counts data. Total read-counts per library (A). Boxplot of transformed data (B). PCA analyses (C). Scatter plot of the first two samples (D).

**(E)** Number of DEG between WT and *Txnip* KO MEF, iPS, and EB samples.

**(F)** Scatter plots for differential expression analysis using DESeq2 in WT and *Txnip* KO iPS and EB samples

**(G-H)** Gene ontology (GO) enrichment analysis differentially expressed genes in WT and *Txnip* KO iPS samples (G) and WT and *Txnip* KO EB samples (H). All differentially expressed genes were subjected to GO analysis – the top enriched terms are shown.

**
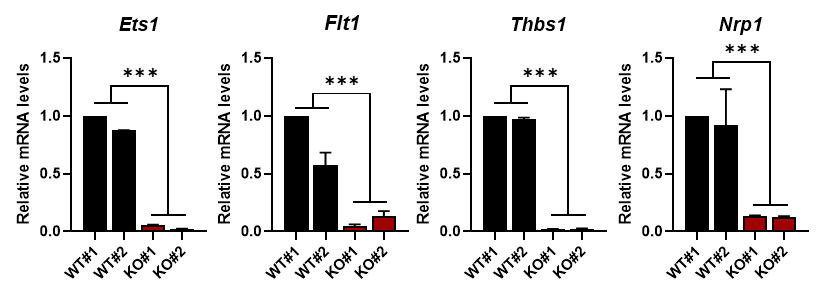
**

**Supplementary Fig. 4** Genes related to defect in *Txnip* KO teratoma formation.

The mRNA expression levels of genes of cluster 7 (*Ets1* and *Flt1)* and cluster 8 *(Thbs1* and *Nrp1)*, which are mostly related to angiogenesis and further more generation of tumor (n=3) Presented as means ± SEM. (*** *p* ≤ 0.001)

**
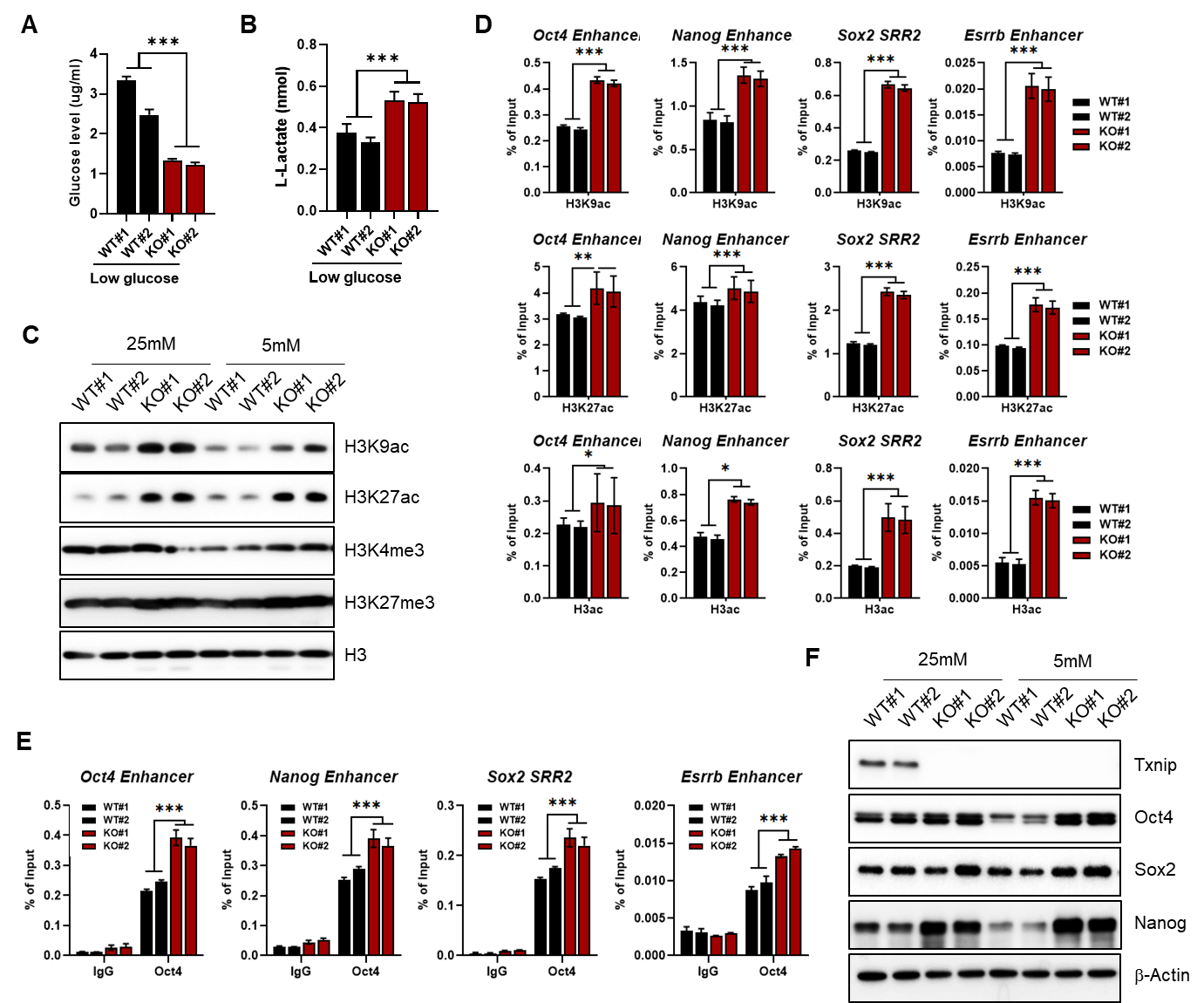
**

**Supplementary Fig. 5** Pluripotency is maintained through low glucose conditions in *Txnip* KO iPSCs.

**(A-B)** Txnip loss elevates glucose metabolism and increases the production of by-products even under low glucose conditions; *Txnip* KO iPSCs showed relatively lower glucose level (A) and higher L-lactate levels (B) in culture media. (n=3) Presented as means ± SEM. (*** *p* ≤ 0.001)

**(C)** The protein levels of histone acetylation in high (25mM) and low glucose (5mM) conditions were assessed by using indicated antibodies. *Txnip* KO iPSCs showed elevated histone acetylation levels in both conditions. Expression was detected by indicated antibodies; H3 was used as a control.

**(D)** ChIP-qPCR analysis of H3K9ac, H3K27ac, and H3ac on active PSC gene regions in WT and *Txnip* KO iPSCs under low glucose conditions. (n=3) Presented as means ± SEM. (* *p* ≤ 0.05, ** *p* ≤ 0.01, *** *p* ≤ 0.001)

**(E)** ChIP-qPCR analysis of Oct4 on active PSC gene regions in WT and *Txnip* KO cells under low glucose conditions. (n=3) Presented as means ± SEM. (*** *p* ≤ 0.001)

**(F)** The protein levels of Txnip, Oct4, Sox2, and Nanog in high (25mM) and low glucose (5mM) conditions were assessed by using indicated antibodies. *Txnip* KO iPSCs still expressed comparable level of pluripotency markers under low glucose conditions. Expression was detected by indicated antibodies; β-Actin was used as an internal control.

**
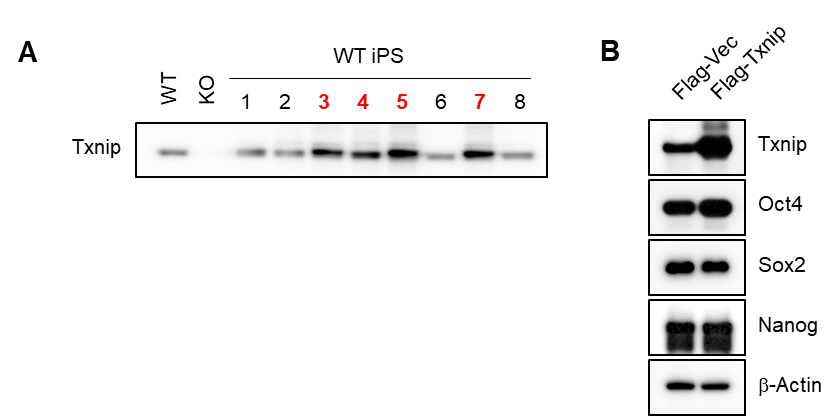
**

**Supplementary Fig. 6** Generation of Txnip overexpressed iPSCs.

**(A)** Western blot analysis of Txnip to verify Txnip overexpression in WT-Flag tagged Txnip-iPSCs. Clones number 3,4,5, and 7 were verified to have Txnip overexpression.

**(B)** The protein levels of Txnip, Oct4, Sox2, and Nanog in Flag-vector and Flag-Txnip overexpressed iPSCs line#5. Expression was detected by indicated antibodies; β-Actin was used as an internal control.


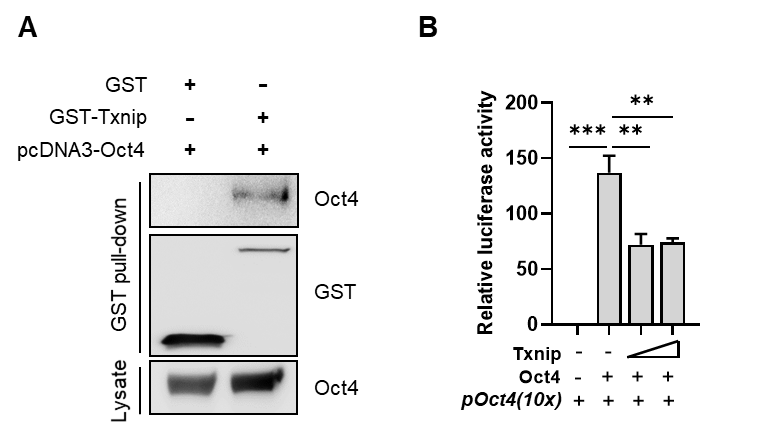


**Supplementary Fig. 7** Txnip directly interacts with Oct4 and inhibits its activity in 293T cells.

**(A)** Glutathione S-transferase (GST) pull-down assay for GST-Txnip and Oct4 proteins. **(B)** Reduction of Oct4–mediated transactivation by Txnip. (n=3) Presented as means ± SEM. (** *p* ≤ 0.01, *** *p* ≤ 0.001)

**Supplementary Table 2.** Primers for RT-qPCR and ChIP-qPCR.

| **qPCR** | | | | |
| --- | --- | --- | --- | --- |
|  | **Forward** | **Reverse** | | **Ref.** |
| *Gapdh* | AGTGTTTCCTCGTCCCGTAG | GCCGTGAGTGGAGTCATACT | | Designed |
| *Txnip* | CCGGACGGGTAATAGTGGAA | ATCTCGTTCTCACCTGCTGT | | Designed |
| *Oct4* | TTGCAGCTCAGCCTTAAGAAC | TCATTGTTGTCGGCTTCCCT | | ^1^ |
| *Sox2* | ACGGCAGCTACAGCATGA | GACGTCGTAGCGGTGCAT | | ^1^ |
| *Nanog* | CCAGGTTCCTTCCTTCTTCC | GGTGAGATGGCTCAGTGGAT | | ^2^ |
| *Esrrb* | AGTACAAGCGACGGCTGGAT | CCTAGTAGATTCGAGACGATCTTAGTCA | | ^3^ |
| *Klf4* | GAAGACCAGGATTCCCTTGA | CCAAGCACCATCATTTAGGC | | ^4^ |
| *Nestin* | CTGCAGGCCACTGAAAAGTT | GACCCTGCTTCTCCTGCTC | | Designed |
| *Bmp4* | TTCCTGGTAACCGAATGCTGA | CCTGAATCTCGGCGACTTTTT | | Designed |
| *Noggin* | GCCAGCAGTATCTACACATCC | GCGTCTCGTTCAGATCCTTCTC | | Designed |
| *Gata4* | ATCAACCGGCCCCTCATTAA | GGGCTTCCGTTTTCTGGTTT | | Designed |
| *Sox17* | GCACAGCAGAACCCAGATCT | CCGGTACTTGTAGTTGGGGT | | Designed |
| *Sma* | ACTGGGACGACATGGAGAAG | GGAAGCATAGAGGGACAGCA | | Designed |
| *T* | ACCCAGCTCTAAGGAACCAC | GCTGGCGTTATGACTCACAG | | Designed |
| *Thbs1* | TGGCCAGCGTTGCCA | TCTGCAGCACCCCCTGAA | | ^5^ |
| *Nrp1* | CGTGGAAGTAATTGATGGGGAG | CATAGCGGATGGAAAACCCTG | | #MP208857 |
| *Ets1* | TCCTATCAGCTCGGAAGAACTC | TCTTGCTTGATGGCAAAGTAGTC | | ^6^ |
| *Flt1* | CCTCACTGCCACTCTCATTGTA | ACAGTTTCAGGTCCTCTCCTT | | ^7^ |
| *Acaca* | GTTCTGTTGGACAACGCCTTCAC | GGAGTCACAGAAGCAGCCCATT | | #MP200195 |
| *Acacb* | AGAAGCGAGCACTGCAAGGTTG | GGAAGATGGACTCCACCTGGTT | | #MP200196 |
| *Acly* | ACCGGCAAAGAACTCCTGTA | ATGTCCCAGGCGAGGTTTTA | | Designed |
| *Acss1* | GCAGGCTATCTACTGTATGCCG | AGGACTGTGGTAGCTCCATTGC | | #MP200286 |
| *Acss2* | CTCCATTGTGTTTGCAGGCT | GTCATTCATGCCCAGCTCTG | | Designed |
| *Hk1* | CACCGGCAGATTGAGGAAAC | CTCAGCCCCATTTCCATCTCT | | ^8^ |
| *Hk2* | AGCTGCTGTTCCAAGGGAAACTCA | GTAGGCCTTCTGAATTCCGTCCTT | | ^8^ |
| *Ldha* | CAAACTCAAGGGCGAGATG | GTTCGCAGTTACACAGTAGTC | | ^9^ |
| *Ldhb* | TGGACAAGTGGGTATGGC | TTTTCGGAGTCTGGAGGA | | Designed |
| *Pdha* | TTCATCGGCTAGAAGAGGGC | TAGGCAGTGATGAGGTGGTC | | Designed |
| *Pdhb* | GTGCAGTTGACAGTTCGTGA | GGGGTGTCGATGATCCTCTT | | Designed |
| *Pkm2* | ACTTGCAGCTATTCGAGGAACTCCG | GGGATTTCGAGTCACGGCAATGAT | | ^8^ |
| **ChIP-qPCR** | | | | |
|  | **Forward** | **Reverse** | |  |
| *Oct4* Enhancer | CTTGAACTGTGGTGGAGAGTGCTG | TAAGGAAGGGCTAGGACGAGAGG | | ^3^ |
| *Nanog* Enhancer | GTCCCCGCTCCTTTTCAGCACTAACCATAC | CGGTTTGAATAGGGAGGAGGGCGTCT | | ^3^ |
| *Sox2 SRR2* | AGTCCAAGCTAGGCAGGTTCCCCT | TGCCCGAGCCCGGGAAATTCTTTT | | Designed |
| *Esrrb Enhancer* | TGAGCTATCAAGTCATTGGCAAAGAGGACA | TTGTTCCGGTCACGTTGTGGGTTCTAT | | ^10^ |
| **shRNAs** | | | | |
| *Txnip* shRNA#1 | pLKO.1 *Txnip* shRNA | | TRCN0000347096 |  |
| *Txnip* shRNA#2 | pLKO.1 *Txnip* shRNA | | TRCN0000182360 |  |

**Reference**

1. Simandi, Z. et al. OCT4 Acts as an Integrator of Pluripotency and Signal-Induced Differentiation. *Mol Cell* **63**, 647-661 (2016).

2. Shigeta, M. et al. Maintenance of pluripotency in mouse ES cells without Trp53. *Sci Rep* **3**, 2944 (2013).

3. Kwak, S. et al. Zinc finger proteins orchestrate active gene silencing during embryonic stem cell differentiation. *Nucleic Acids Res* **46**, 6592-6607 (2018).

4. Aksoy, I. et al. Klf4 and Klf5 differentially inhibit mesoderm and endoderm differentiation in embryonic stem cells. *Nat Commun* **5**, 3719 (2014).

5. Liu, Z. et al. Thrombospondin-1 (TSP1) contributes to the development of vascular inflammation by regulating monocytic cell motility in mouse models of abdominal aortic aneurysm. *Circ Res* **117**, 129-41 (2015).

6. Li, K. et al. Ets1-Mediated Acetylation of FoxO1 Is Critical for Gluconeogenesis Regulation during Feed-Fast Cycles. *Cell Rep* **26**, 2998-3010 e5 (2019).

7. Hedlund, E.M. et al. Tumor cell-derived placental growth factor sensitizes antiangiogenic and antitumor effects of anti-VEGF drugs. *Proc Natl Acad Sci U S A* **110**, 654-9 (2013).

8. Kim, H. et al. Core Pluripotency Factors Directly Regulate Metabolism in Embryonic Stem Cell to Maintain Pluripotency. *Stem Cells* **33**, 2699-711 (2015).

9. Nishimura, K. et al. A Role for KLF4 in Promoting the Metabolic Shift via TCL1 during Induced Pluripotent Stem Cell Generation. *Stem Cell Reports* **8**, 787-801 (2017).

10. Wu, Q. et al. Sall4 interacts with Nanog and co-occupies Nanog genomic sites in embryonic stem cells. *J Biol Chem* **281**, 24090-4 (2006).
